# Buried SWCNTs Interlayer Promotes Hole Extraction and Stability in Inverted CsPbI_2.85_Br_0.15_ Perovskite Solar Cells

**DOI:** 10.3390/molecules30173535

**Published:** 2025-08-29

**Authors:** Fangtao Yu, Dandan Chen, He Xi, Wenming Chai, Yuhao Yan, Weidong Zhu, Dazheng Chen, Long Zhou, Yimin Lei, Chunfu Zhang

**Affiliations:** 1 State Key Laboratory of Wide Bandgap Semiconductor Devices and Integrated Technology, School of Advanced Materials and Nanotechnology, Xidian University, Xi’an 710071, China; 24141213565@stu.xidian.edu.cn (F.Y.); c15029352460@163.com (D.C.); 23141214078@stu.xidian.edu.cn (Y.Y.); zhoulong@xidian.edu.cn (L.Z.); 2 Faculty of Integrated Circuit, Xidian University, Xi’an 710071, China; chaiwenming@xidian.edu.cn (W.C.); wdzhu@xidian.edu.cn (W.Z.); dzchen@xidian.edu.cn (D.C.)

**Keywords:** all-inorganic PSCs, inverted structure, single-walled carbon nanotubes, buried interface modification

## Abstract

Inverted (p-i-n) CsPbI_x_Br_3−x_ (x = 0~3) perovskite solar cells (PSCs) are of growing interest due to their excellent thermal stability and optoelectronic performance. However, they suffer from severe energy level mismatch and significant interfacial energy losses at the bottom hole transport layers (HTLs). Herein, we propose a strategy to simultaneously enhance the crystallinity of CsPbI_2.85_Br_0.15_ and facilitate hole extraction at the HTL/CsPbI_2.85_Br_0.15_ interface by incorporating semiconducting single-walled carbon nanotubes (SWCNTs) onto [2-(3,6-dimethoxy-9H-carbazol-9-yl)ethyl] phosphonic acid (MeO-2PACz) HTL. The unique electrical properties of SWCNTs enable the MeO-2PACz/SWCNT HTL to achieve high conductivity, optimal energy level alignment, and an adaptable surface. Consequently, the defect density is reduced, hole extraction is accelerated, and interfacial charge recombination is effectively suppressed. As a result, these synergistic benefits boost the power conversion efficiency (PCE) from 15.74% to 18.78%. Moreover, unencapsulated devices retained 92.35% of their initial PCE after 150 h of storage in ambient air and 89.03% after accelerated aging at 85 °C for 10 h. These findings highlight the strong potential of SWCNTs as an effective interlayer for inverted CsPbI_2.85_Br_0.15_ PSCs and provide a promising strategy for designing high-performance HTLs by integrating SWCNTs with self-assembled monolayers (SAMs).

## 1. Introduction

All-inorganic cesium lead halide perovskites (CsPbX_3_, X = I, Br, Cl) have attracted growing research interest recently due to their superior thermal stability and optoelectronic properties [1,2,3,4]. Specially, the maximum power conversion efficiency (PCE) of all-inorganic perovskite solar cells (PSCs) has reached 22.20% [5], demonstrating their significant potential for both single-junction and front-cell configurations of tandem solar cell applications. In inorganic PSCs, the mixed-halide strategy is frequently employed to optimize the optical bandgap and phase stability of CsPbI_x_Br_3−x_ (x = 0~3) perovskites [6,7,8,9]. Among them, the representative perovskites CsPbI_2_Br and CsPbIBr_2_ stand out for their well-balanced phase stability and photovoltaic performance, achieving maximum efficiency rates of 18.39% and 12.8%, respectively [10,11]. Despite recent progress, the efficiency of mixed-halide CsPbI_x_Br_3−x_ PSCs still lags behind that of their organic–inorganic hybrid counterparts and certain pure-halide compositions, such as CsPbI_3_. The efficiency gap can be primarily attributed to the substantial open-circuit voltage (V_OC_) loss, reflecting severe nonradiative recombination losses both within the perovskite bulk and at the interfaces with the charge transport layers (CTLs) [9,12,13,14]. These issues are particularly pronounced in CsPbI_x_Br_3−x_ PSCs employing an inverted p-i-n planar configuration.

In typical inverted PSCs, the perovskite layer is directly deposited onto the hole transport layers (HTLs). Benefitting from the absence of hygroscopic and unstable dopants, both the bottom HTLs and top electron transport layers (ETLs) show enhanced stability [15,16]. Hence, inverted PSCs offer notable advantages, including superior enduring stability, reduced hysteresis, and simplified integration into tandem devices [16,17,18]. To date, their certified efficiency has reached 27.00%, surpassing that of regular n-i-p configurations. Additionally, inverted PSCs also set the highest PCE records in both all-perovskite and silicon/perovskite tandem solar cells [19,20]. However, the current highest reported efficiency of inverted CsPbI_x_Br_3−x_ PSCs is still far below the Shockley–Queisser (S-Q) limit, mainly due to the poor morphology of polycrystalline CsPbI_x_Br_3−x_ films and ineffective carrier transport caused by interface defects and energy-level mismatch at the HTL/perovskite buried interface [21].

Rational design of the buried interface is essential for high-performance inverted CsPbI_x_Br_3−x_ PSCs, as it simultaneously regulates the crystallinity of both the underlying interfacial and bulk perovskite regions, facilitates efficient charge extraction, and suppresses nonradiative losses. Phosphonic acid self-assembled monolayers (SAMs) based on a carbazole core, including [2-(3,6-dimethoxy-9H-carbazol-9-yl)ethyl]phosphonic acid (MeO-2PACz) and [4-(3,6-dimethyl-9H-carbazol-9-yl)butyl]phosphonic acid (Me-4PACz), have been put forward as viable alternatives to conventional polymer and metal oxide HTLs in inverted PSCs because of their superior hole selectivity and extraction capabilities [22,23]. However, due to their amphipathic nature, phosphonic acid self-assembled monolayers (SAMs) often suffer from severe inferiority of agglomeration and poor surface morphology, especially under thermal stress, leading to serious defects and leakage current at the interface and damaging the PSCs’ performance and stability. Thus, regulating the surface and electronic properties of SAMs is critical for controlling perovskite film crystallization and optimizing the performance of inverted all-inorganic PSCs. Our group previously tried to regulate the orientation of SAM molecules by introducing a rare-earth chloride (TbCl_3_) doping strategy, enabling the conformal growth of Me-4PACz on FTO substrates. This approach simultaneously reduced energy level mismatch, ultimately improving the efficiency of PSCs from 15.34% to 18.68% [24]. In addition, our group also applied CsF to modify the buried interface. CsF effectively promoted more uniform spreading and the formation of a dense, flat perovskite film while optimizing the energy level alignment between the HTL and the perovskite layer. As a result, the PCE of the PSCs increased from 16.15% to 18.24% [25].

Single-walled carbon nanotubes (SWCNTs) have attracted great attention, serving as transparent conductive electrodes, charge transporters, and interlayers for highly efficient PSCs due to their remarkable photoelectronic and mechanical properties, as well as chemical inertness [26,27]. Zhang et al. employed SWCNTs as both the front and rear electrodes [28]. By precisely tuning the thickness of the SWCNT layers, they achieved a balanced optimization of optical transmittance and sheet resistance, leading to a front-side efficiency of 19.14% and an equivalent conversion efficiency of total bifacial power generation of 31.82%. Sun et al. increased the PCE of carbon-based CsPbBr_3_ PSCs from 6.58% to 10.47% by integrating SWCNTs and WO_3_ as a composite hole transport layer (HTL) [29]. This improvement is attributed to the incorporated SWCNTs, which enhance hole transport to the carbon back electrode and passivate metal ion defects within the CsPbBr_3_ film. Habisreutinger et al. designed a double-layer HTL structure comprising SWCNTs/(PMMA or spiro-OMeTAD) [30]. Leveraging the exceptionally high hole mobility of SWCNTs and their superior charge transfer kinetics with the perovskite layer, this configuration enables rapid extraction of holes from the perovskite. This significantly reduces hole accumulation at the interface, thereby substantially suppressing interfacial recombination. Consequently, PSCs employing SWCNTs/PMMA and SWCNTs/spiro-OMeTAD HTLs achieved PCEs of 17.4% and 20.0%, respectively. These studies have demonstrated that SWCNTs have shown considerable potential in conventional structures for enhancing both the crystalline quality of perovskite thin films and the corresponding photovoltaic performance, which has inspired us to explore the application of SWCNTs serving as a modification layer in inverted-structure PSCs.

In this study, we utilized SWCNTs to modify the MeO-2PACz/CsPbI_2.85_Br_0.15_ interface. The SWCNTs decreased the hydrophilicity of MeO-2PACz and enhanced the crystallization of CsPbI_2.85_Br_0.15_ films. Moreover, it could improve the energy level alignment between MeO-2PACz and CsPbI_2.85_Br_0.15_, resulting in the promoted carrier extraction at the interface. As a result, the inverted CsPbI_2.85_Br_0.15_ PSCs achieved an optimal efficiency of 18.78% (compared to the unmodified device, which showed a PCE of 15.74%) and superior stability in ambient air and under 85 °C. This method facilitates the preparation of efficient and stable CsPbI_2.85_Br_0.15_ PSCs.

## 2. Results and Discussion

**Figure 1:**
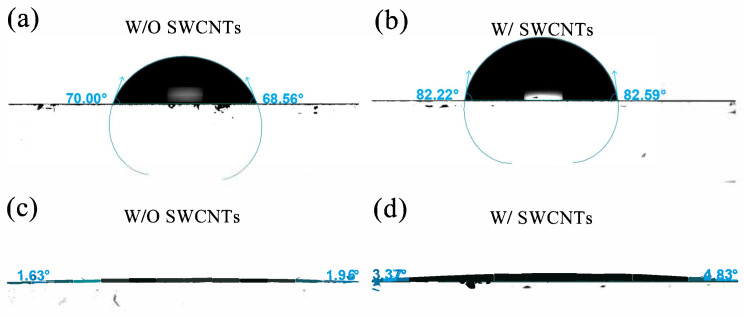
Contact angle images of MeO-2PACz films: (**a**,**b**) water and (**c**,**d**) DMSO/DMF mixed solvent on pristine and SWCNTs modification.

Owing to the unique physicochemical characteristics of SWCNTs, we employed SWCNTs to alter the MeO-2PACz/CsPbI_2.85_Br_0.15_ interface properties to enhance PSC performance. The effect of SWCNTs on the performance of MeO-2PACz HTL was systematically investigated. Atomic force microscopy (AFM) images of the MeO-2PACz and MeO-2PACz/SWCNTs films are shown in Figure S1 (Supplementary Materials). It can be observed that MeO-2PACz films with and without SWCNTs display uniform and compact surface morphology. The root mean square (RMS) roughness values of the MeO-2PACz HTL and MeO-2PACz/SWCNTs films were 26.8 nm and 27.4 nm, respectively, indicating that the addition of SWCNTs had no discernible impact on the surface morphology of MeO-2PACz HTL. The effect of SWCNTs on the surface wettability of MeO-2PACz was evaluated by contact angle (CA) measurement. As shown in Figure 1a,b, the water contact angle (WCA) values were 70.00° (L)/68.56° (R) for MeO-2PACz and 82.22° (L)/82.59° (R) for MeO-2PACz/SWCNTs. The increased WCA indicates reduced surface wettability. Compared to MeO-2PACz films, the increased WCA of SWCNT-modified MeO-2PACz provides a better barrier against the water intrusion, contributing to improved long-term stability. Given that the perovskite was dissolved in a DMSO/DMF mixed solvent during the device preparation process, we also measured the contact angles of this mixed solvent. The obtained values were 1.63° (L)/1.96° (R) for MeO-2PACz and 3.37° (L)/4.83° (R) for MeO-2PACz/SWCNTs, as shown in Figure 1c,d. For a super-solvent-philic HTL, moderately reducing its wettability toward the mixed solvent suppresses excessive heterogeneous nucleation, decreases nucleus density, and provides space for lateral grain growth, thereby facilitating the formation of larger grains [31]. Consequently, this process reduces grain boundary defects and recombination centers, ultimately yielding higher-quality perovskite films and enhanced device performance. These results confirm the same trend of increased contact angles and reduced wettability on SWCNT-modified surfaces.

**Figure 2:**
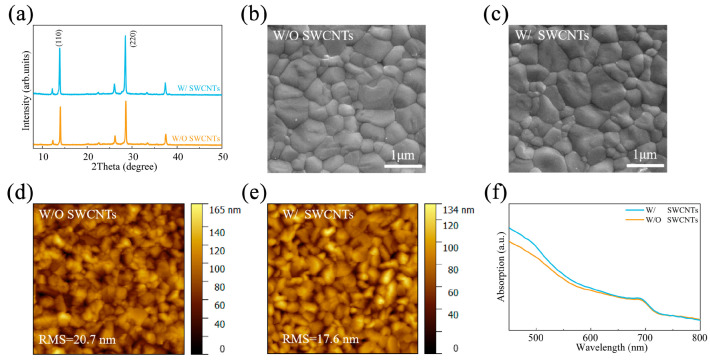
(**a**) XRD patterns. (**b**,**c**) SEM and (**d**,**e**) AFM images of CsPbI_2.85_Br_0.15_ films without and with SWCNTs, respectively. (**f**) UV-visible absorption spectra for pure and SWCNT-modified CsPbI_2.85_Br_0.15_ films.

To create p-i-n structured PSCs, we deposited perovskite films on MeO-2PACz and MeO-2PACz/SWCNTs. We employed X-ray diffraction (XRD), scanning electron microscopy (SEM), atomic force microscopy (AFM), Kelvin probe force microscopy (KPFM), and the ultraviolet-visible (UV-Vis) to further investigate the effects of SWCNT alteration. XRD was used to examine the crystalline structure of CsPbI_2.85_Br_0.15_ films with and without SWCNTs. The XRD peaks at 14.03° and 28.57°, which point to the (110) and (220) crystal planes of CsPbI_2.85_Br_0.15_ material with cubic perovskite phase [2,31], respectively, are similar, as shown in Figure 2a. The SEM images of the CsPbI_2.85_Br_0.15_ films with and without SWCNTs are displayed in Figure 2b,c. The SEM results are consistent with the enhanced crystallinity of CsPbI_2.85_Br_0.15_ film modified with SWCNTs, as evidenced by its significantly higher XRD peak intensity compared to the non-modified sample. The SWCNT-modified CsPbI_2.85_Br_0.15_ film presented larger and more uniform grains, with grain size increasing from 510 nm to 610 nm compared to the pure samples (Figure S2). In the final CsPbI_2.85_Br_0.15_ PSCs, this demonstrates that SWCNT modification can help limit the potential of nonradiative recombination of charge carriers, which can be explained by the fact that intragranular defects and/or grain boundaries are suppressed when SWCNTs are modified. Moreover, the surface morphology and RMS roughness of the perovskite films were investigated using AFM (Figure 2d,e). It was found that the surface roughness of perovskite films with and without SWCNT modification were 17.6 nm and 20.7 nm, respectively. These findings suggest that the surface roughness at the top of perovskite films can be decreased by adding SWCNTs to the MeO-2PACz/CsPbI_2.85_Br_0.15_ interfacial layer, which is beneficial for the interfacial charge transport. We have observed that, compared to the results from our previous work using CsF to modify the buried interface, perovskite film modified with SWCNTs exhibited a much smoother surface [25]. This is likely because SWCNTs are porous and highly permeable. During the spin-coating and annealing processes of the perovskite film, solvents (such as DMF and DMSO) can rapidly and uniformly evaporate through the carbon nanotube. A uniform solvent evaporation rate is a crucial factor in obtaining a pinhole-free and smooth film. Furthermore, KPFM was used to measure the average surface contact potential difference (CPD) on CsPbI_2.85_Br_0.15_ films. The CPD mappings are displayed in Figure S3. Compared to non-modified films, the CPD values of SWCNTs-modified films were comparatively lower. The decreases corresponded to an upward shift in the work function (WF) of the underlying MeO-2PACz/SWCNTs layer, which induced a downward movement of the Fermi level toward the valence band maximum (VBM) at the perovskite/HTL interface. Figure 2f illustrates how the crystallization of the perovskite films causes the absorbance of the SWCNTs-modified films to increase to the 500–800 nm range. Both samples had similar absorption onsets (~723 nm), corresponding to an optical bandgap of 1.72 eV. All these optimizations of perovskite films can directly contribute to high-performance and stable PSCs.

**Figure 3:**
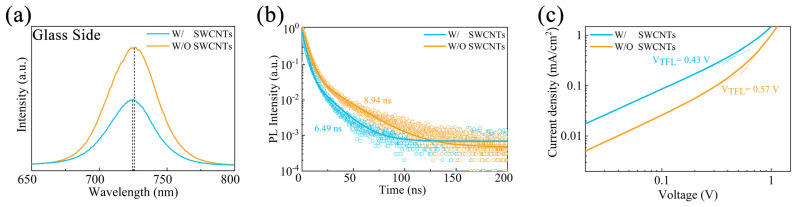
(**a**) PL and (**b**) TRPL of CsPbI_2.85_Br_0.15_ films on FTO/MeO-2PACz without and with SWCNT modification, respectively. (**c**) SCLC curves of CsPbI_2.85_Br_0.15_ films without and with SWCNT modification.

Steady-state photoluminescence (PL) and time-resolved PL (TRPL) spectroscopy of the perovskite films with and without SWCNTs were recorded from the glass side to confirm the impact of SWCNTs on the charge carrier dynamics within the perovskites. To ensure statistical reliability, a PL/TRPL dataset was obtained from 20 independently fabricated samples, and the observed trends were consistent. As seen in Figure 3a, both samples exhibited a comparable PL peak at ~723 nm. However, the SWCNT-modified perovskite films exhibited 45% lower PL intensity than the control, indicating enhanced carrier extraction at the buried interface. This confirms that SWCNTs can enhance perovskite crystallinity and carrier extraction capabilities at the interface [32]. The TRPL spectra of the perovskite films deposited on the MeO-2PACz with and without SWCNTs are shown in Figure 3b, and Table S1 provides a summary of the fitted results. A significant reduction in carrier lifetime was observed at the perovskite/HTL interface. Specifically, compared with the control samples, the fast decay lifetime (*τ*_1_) of the SWCNT-modified CsPbI_2.85_Br_0.15_ films decreased from 4.43 ns to 3.30 ns, and the slow decay lifetime (*τ*_2_) decreased from 22.93 ns to 15.48 ns. The SWCNT-modified CsPbI_2.85_Br_0.15_ films possessed a lower average lifetime of carriers (*τ_ave_* = 6.49 ns) compared with the control perovskite (*τ_ave_* = 8.94 ns), indicating enhanced interfacial hole extraction efficiency. In other words, the modification of SWCNTs not only efficiently reduced nonradiative carrier recombination at the buried interface, but also facilitated carrier transport. Furthermore, using the hole-only device with the structure of FTO/MeO-2PACz/SWCNTs/CsPbI_2.85_Br_0.15_/MoO_3_/Cu, we assessed the trap state density (*n_trap_*) variations by the space-charge limited current (SCLC) method [33,34,35]. The following formula can be used to calculate the *n_trap_* [36]:

(1) $$n_{trap}\;=\;\frac{2\varepsilon_{0}\varepsilon V_{TEL}}{eL^{2}}$$

where *ε*_0_, *ε*, *e*, *V_TEL_*, and *L* are vacuum permittivity (*ε*_0_ = 8.85 × 10^−12^ F·m^−1^), the relative dielectric constant (*ε* = 7.14), the elementary charge of the electron (*e* = 1.6 × 10^−19^ *C*), and the thickness of the perovskite film (*L* = 500 nm), respectively. The *V_TFL_* of the control device was 0.56 V, as can be seen in Figure 3c, but after modification with SWCNTs, it dropped to 0.43 V. The control films exhibited a *n_trap_* of 1.77 × 10^16^ cm^−3^, while the SWCNT-modified films showed a reduced *n_trap_* of 1.36 × 10^16^ cm^−3^. The decrease in *n_trap_* represents fewer defects in the SWCNT-modified films, which is conducive to suppressing the nonradiative recombination of carriers at the internal interface of the device and further increases the V_OC_. Unlike CsF modification, which passivates defects (such as Pb^2+^) at the perovskite/HTL interface through F^−^ to reduce the actual defect density [25], SWCNT modification enables photogenerated holes to be extracted and transported to the HTL more rapidly, significantly shortening their residence time at the interface. This greatly reduces the probability of their capture by defects, thereby resulting in a lower apparent defect density in the measurements. Additionally, we evaluated the humidity stability of the perovskite films by exposing them to an air environment with a relative humidity of 30–40%. As shown in Figure S4, the unmodified perovskite film began to change color at 24 h due to surface degradation caused by moisture and oxygen, forming PbI_2_. The yellowed areas then continued to expand, and they covered most of the film’s surface by 96 h. In contrast, the SWCNT-modified perovskite film remained pristine, with no significant changes observed. The enhanced stability stemmed from the SWCNT-modified MeO-2PACz layer providing superior protection to the perovskite, inhibiting the ingress of moisture and thus improving the film stability.

A PSC device with the structure FTO/MeO2PACz/SWCNTs/CsPbI_2.85_Br_0.15_/PC61BM/C60/BCP/Cu was manufactured to assess the impact of interfacial alteration of SWCNTs on the photovoltaic applications of perovskite cells (Figure S5). Ultraviolet photoelectron spectroscopy (UPS) spectra were further performed to carefully investigate the WF and energy level alignment of MeO-2PACz with and without SWCNT modification. Figure S6 presents the secondary electron cutoffs (*E_cutoff_*) and the valence band maximum onsets (*E_onset_*), from which the WFs of the MeO-2PACz and MeO-2PACz/SWCNTs HTLs were derived to be 4.23 eV and 4.53 eV, respectively, using the following formula [37]:

(2) $$WF=21.22ev\;-\;E_{cutoff}$$

**Figure 4:**
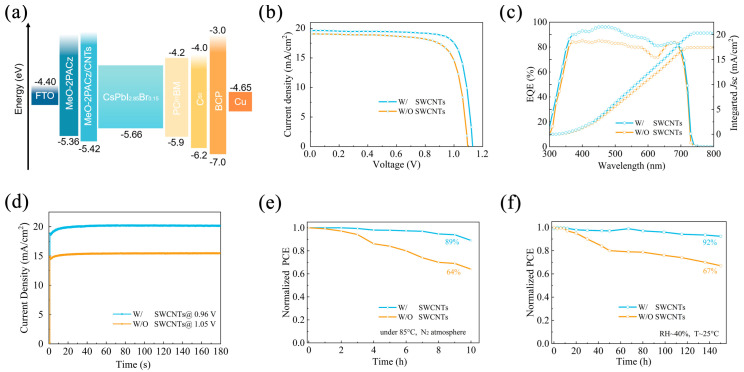
(**a**) Energy level alignment, (**b**) J-V curves, (**c**) EQE spectra, (**d**) stabilized current outputs, (**e**) thermal stability, and (**f**) long-term stability of CsPbI_2.85_Br_0.15_ PSCs without and with SWCNT modification, respectively.

According to the following formula [38]:

(3) $$VBM=21.22ev\;-\;(E_{cutoff}-E_{\mathrm{oneset}})$$

the corresponding VBMs were calculated as −5.36 and −5.42 eV, respectively. These values correspond to the CPD results. Figure 4a shows a schematic of the energy band arrangement of the SWCNT-optimized CsPbI_2.85_Br_0.15_ PSCs. SWCNT modification effectively reduced the valence band maximum (VBM) mismatch between MeO-2PACz and the CsPbI_2.85_Br_0.15_ layer. This facilitates charge extraction and transport at the MeO-2PACz/CsPbI_2.85_Br_0.15_ interface [39,40], suppresses charge recombination, and further enhances the V_OC_ and fill factor (FF) of the devices. Consequently, the PCE was ultimately improved [41,42,43]. To provide a reliable baseline for evaluating the impact of SWCNT modification, the performance of the control devices was compared with our group’s previous results on the identical CsPbI_2.85_Br_0.15_ system [25]. The photovoltaic parameters were comparable, with variations remaining within the typical batch-to-batch deviation for solution-processed perovskite devices (Table S2). This confirms that the significant enhancement observed in the SWCNT-modified devices can be directly attributed to the SWCNT modification strategy. The photocurrent density–voltage (J-V) curves of the PSCs with and without SWCNTs are displayed in Figure 4b. Table S3 provides an overview of the PV parameters. The devices that were modified with SWCNTs showed increases from 15.74 to 18.78% in PCE, from 1.09 to 1.12 V in V_OC_, from 19.09 to 20.83 mA cm^−2^ in J_SC_, and from 75.66 to 80.81% in FF. Figure S7 shows reduced dark current density in SWCNT-modified PSCs compared to the control. This indicates lower defect density and suppressed carrier leakage at interfaces, consistent with passivated grain boundaries and optimized charge transport. The photovoltaic parameters of 18 independent devices were statistically analyzed, as shown in Figure S8. Compared to the control sample, SWCNT-modified PSCs had greater average values of V_OC_, J_SC_, FF, and PCE, with narrow dispersion and strong reproducibility. This is consistent with the trend observed in the JV curve.

Next, we tested the integrated photocurrent density and external quantum efficiency (EQE) of the devices with and without the addition of SWCNTs. As shown in Figure 4c, all films had a photo response cutoff of roughly 720 nm. For the SWCNT-modified CsPbI_2.85_Br_0.15_ PSCs, the integral J_SC_ value was 20.3 mA cm^−2^, whereas for the control CsPbI_2.85_Br_0.15_ PSCs, it was 17.4 mA cm^−2^. In line with the JV results in Figure 4b, the devices with SWCNTs demonstrated increased photoelectric conversion efficiency throughout the full response wavelength range. Figure 4d shows the steady-state photocurrent density of the best device without and with SWCNTs measured at maximum power point. The stabilized efficiencies matched the findings from the J-V measurements. This might result in less nonradiative recombination and more charge transfer capacity due to optimized energy band alignment and reduced defect density. One essential component for the commercialization of CsPbI_2.85_Br_0.15_ PSCs is their stability. To evaluate this critical aspect, we subjected the devices to thermal stability testing. After 10 h of continuous heating, SWCNT-modified devices retained 89.03% of their initial PCE (Figure 4e), while control devices degraded to 64.06%. Furthermore, as Figure 4f illustrates, we looked into the ambient stability of the PSC devices enhanced with SWCNTs. After 150 h of storage in an air environment (RH~40%, T~25 °C), the modified devices retained 92.35% of their initial efficiency, but the control group only maintained 67.00% of its initial efficiency. The stabilization of PSCs was greatly enhanced by the modification of SWCNTs, primarily through the suppression of device defects and stabilization of perovskite crystals. Moreover, the reduced wettability toward water effectively prevented bottom-up ingress of water and oxygen. Thus, the interface modification of MeO-2PACz/CsPbI_2.85_Br_0.15_ could enhance the commercialization potential of resulting perovskite solar cells.

**Figure 5:**
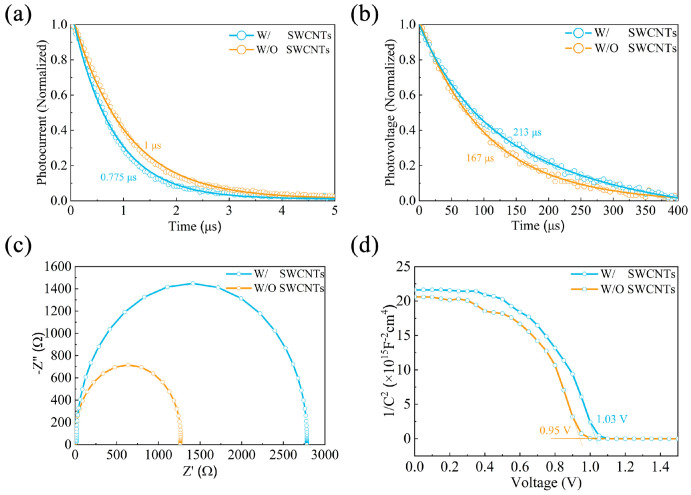
(**a**) TPC, (**b**) TPV (**c**) Nyquist plots, and (**d**) M-S curves for CsPbI_2.85_Br_0.15_ PSCs based on MeO2PACz/SWCNTs and MeO-2PACz HTL, respectively.

To investigate the impact of SWCNTs on the dynamics of carrier complexes in the devices and the defect states in CsPbI_2.85_Br_0.15_ thin films, we carried out several electrical characterizations. A study on the interfacial transport of charge carriers within PSCs was performed using transient photocurrent (TPC) characterization (Figure 5a). The TPC results demonstrate that the PSCs modified with SWCNTs had a faster charge transport time (0.775 μs) than the control PSCs (1 μs). Faster charge extraction and transport are implied by the shorter charge transport time. The transient photovoltage (TPV) curve in Figure 5b shows that the charge recombination lifetime of the SWCNT-modified device increased to 213 μs in comparison with the 167 μs of the control ones, representing the reduction of the defect-state density within the perovskite. The extended lifetime implies that reduced carrier recombination and more effective carrier extraction contributed to the improved performance of CsPbI_2.85_Br_0.15_ PSCs with SWCNTs. To learn more about the altered charge transport and recombination inside the PSCs caused by the SWCNTs, electrochemical impedance spectroscopy (EIS) measurement was carried out (Figure 5c). CsPbI_2.85_Br_0.15_ PSCs prepared with SWCNT-modified MeO-2PACz exhibited increased recombination resistance (R_rec_) from 1262 to 2783 Ω in comparison with the control sample, indicating that the carrier recombination was suppressed at the MeO-2PACz/CsPbI_2.85_Br_0.15_ interface. This improvement is primarily attributed to the suppression of interfacial defects at the MeO-2PACz/CsPbI_2.85_Br_0.15_ interface, as well as the reduction of bulk defects within the perovskite layer after SWCNT modification. To validate such a hypothesis, the built-in potential (V_bi_) of PSCs was extracted from Mott–Schottky curves. The Mott–Schottky method was used to verify the V_OC_ enhancement. The test results (Figure 5d) show that the V_bi_ of the SWCNT-modified PSCs was 1.13 V, significantly higher than the control PSCs (V_bi_ = 1.085 V), and this trend is also consistent with the J-V test results. The enhanced V_bi_ can explain FF and V_OC_ enhancement in PV devices. Therefore, the results of the above device characterization consistently show that SWCNTs inhibit nonradiative recombination with a significant contribution, resulting in enhanced charge transfer capability and improved PV performance.

## 3. Experimental Section

### 3.1. Materials and Reagents

Cesium iodide (CsI, 99.999%), lead iodide (PbI_2_, 99.999%), dimethylammonium iodide (DMAI, 99.5%), lead bromide (PbBr_2_, 99.999%), [6,6]-Phenyl C61 butyric acid methyl ester (PC61BM, 99%), bathocuproine (BCP, 99.5%), C60 (99%) were obtained from Xi’an Polymer Light Technology Corp (Xi’an, China). [2-(3,6-dimethoxy-9H-carbazol-9-yl)ethyl]phosphonic acid (MeO-2PACz, 99.8%) was obtained from TCI Inc (Shanghai, China). N, N-dimethylformamide (DMF, 99.8%), dimethyl sulfoxide (DMSO, 99.9%), chlorobenzene (CB, 99.8%), isopropyl alcohol (IPA, 99.9%), and ethanol (99.7%) were purchased from Sigma Aldrich (Shanghai, China). Patterned fluorine-doped tin oxide (FTO) glass was obtained from Yingkou OPV Tech New Energy Co., ltd. (Yingkou, China). The SWCNTs were synthesized with reference to previous work [44].

### 3.2. Preparation of the Solution

First, 1 mg of MeO-2PACz was dissolved in 1 mL of alcohol and stirred on a stirring table for 4 h to allow for a full reaction. SWCNTs were mixed with CB at a 2:1 ratio. Then, 1 mL of SWCNT solution was dissolved in 1 mL of absolute ethanol and stirred for 3 h to prepare the SWCNT modification layer solution. The solution of perovskite precursor consisted of 168 mg of PbBr_2_, 156 mg of CsI, 280 mg of PbI_2_, and 120 mg of DMAI in DMF/DMSO (*v/v* = 9:1). Then, 10 mg/mL PC61BM/CB mixed solution was obtained to prepare the ETL.

### 3.3. Device Fabrications

FTO glass substrate with dimensions of 2 cm × 2.5 cm was treated with Decon 90, deionized water, acetone, and ethanol sequentially using ultrasonic cleaner for 20 min, followed by treatment with UV-ozone for 15 min. Then, the MeO-2PACz precursor solution was spin-coated on the FTO substrate at 3000 rpm for 30 s and subsequently annealed on a hot plate in an N_2_-filled glovebox at 100 °C for 10 min. After cooling the annealed MeO-2PACz films, the SWCNTs were applied by spin-coating at 4000 rpm for 30 s. Next, the perovskite layer was spin-coated on a MeO-2PACz-FTO substrate for 30 s at a rate of 4000 r/min after starting at 1000 rpm for 5 s. The fabricated perovskite substrates were immediately transferred into the N_2_-filled glovebox after annealing at 200 °C for 5 min. Then, the PC61BM solution was spin-coated on the obtained perovskite films at 4000 rpm for 30 s. Finally, 30 nm C60, 6 nm BCP, and 100 nm Cu electrodes on the top of the cell were prepared by thermal evaporation to complete the device with an active area of 0.07 cm^2^.

### 3.4. Characterization

Using Bruker D8 Advance XRD equipment, Cu Kα (λ = 1.5405 Å) was evaluated at 40 kV and 200 mA for XRD patterns. Using FEI version 460, SEM measurements were performed at various magnifications. A spectrophotometer called Lambda 950 was used to obtain the UV-Vis spectra. The FluoTime 300 spectrometer was used to measure both steady-state photoluminescence (PL) and time-resolved photoluminescence (TRPL). Using a Bruker Dimension Icon microscope, AFM and KPFM pictures were captured. A Keithley 2450 source meter was used to measure the light J-V curves in simulated AM 1.5 G illumination. Before measurement, a silicon cell was used to calibrate the solar intensity. A reverse scan was conducted from 1.3 V to −0.2 V. A Keithley 2450 source meter was used to measure the dark J-V and SCLC in the absence of light. Hole-only devices were necessary for EQE testing. The Biolin Theta Flex model was employed by the contact angle test apparatus. Using a sampling resistor and a pulsed laser with a wavelength of 532 nm and 420 nm, respectively, the samples were stimulated to record the TPC and TPV signals on a digital oscilloscope (Tektronix, MSO5204B, Beaverton, OR, USA). On a Thermo Scientific Escalab 250 Xi (Waltham, MA, USA), an ultraviolet photoelectron spectroscopy (UPS) measurement was carried out. UPS used He I radiation lines with 21.22 eV of energy. Using monochromatic light with a wavelength range of 300 to 800 nm, a SCS10-X150 system was used to obtain the external quantum efficiency curves (EQE). Using an electrochemical workstation in the dark, EIS and M-S measurements were performed on the perovskite solar cells.

## 4. Conclusions

In summary, we have proposed an effective modification method for SAM by introducing SWCNTs between the MeO-2PACz and perovskite layers in planar p-i-n CsPbI_2.85_Br_0.15_ PSCs. The SWCNT modification enhanced the hydrophobicity of the MeO-2PACz layer and decreased the wettability of the perovskite precursor solution, thereby enabling enlarged perovskite grain size with enhanced crystalline quality. The unique electrical properties of SWCNTs enabled the MeO-2PACz/SWCNTs HTL to achieve optimal energy level alignment by minimizing the VBM offset between MeO-2PACz and the CsPbI_2.85_Br_0.15_ layer, which further suppressed interfacial charge recombination and facilitated efficient hole extraction and transport at the interface. As a consequence, the PCE of SWCNT-modified PSCs was significantly improved from 15.74% to 18.78%, and the FF increased from 75.66% to 80.81%. Meanwhile, unencapsulated SWCNT-modified PSCs maintained 92.35% of their initial PCE after 150 h of storage in ambient air and 89.03% after accelerated aging at 85 °C for 10 h, exhibiting enhanced ambient stability and thermal stability.

## Data Availability

Data are available upon request.

## Supplementary Materials

The following supporting information can be downloaded at https://www.mdpi.com/article/10.3390/molecules30173535/s1: Figure S1: AFM images of MeO-2PACz films (a) without and (b) with SWCNTs modification; Figure S2: Statistical distribution of grain sizes in perovskite films (a) without and (b) with SWCNTs modification; Figure S3: KPFM images of (a) pristine and (b) SWCNTs-modified perovskite films; Figure S4: Degradation comparison of CsPbI_2.85_Br_0.15_ films with and without SWCNTs modification after 96 h exposure to ambient air (30~40% RH); Figure S5: Device architecture of the p-i-n structured device; Figure S6: UPS spectra of MeO-2PACz films with and without SWCNTs modification, along with the estimated E_onset_ and E_cutoff_ values obtained from linear fittings; Figure S7: Dark current characteristics of devices with and without SWCNTs modification; Figure S8: Statistical photovoltaic parameters of the control and SWCNTs-modified PSCs: (a) V_oc_, (b) PCE, (c) FF and (d) J_sc_; Table S1: TRPL parameters of CsPbI_2.85_Br_0.15_ PSCs with and without SWCNTs modification; Table S2: The parameters of the best-performing control devices (CsPbI_2.85_Br_0.15_) in the two studies. Table S3:Photovoltaic performance parameters of the best-performing CsPbI_2.85_Br_0.15_ PSCs without and with SWCNTs modification.
