# Supplementary material for: Buried SWCNTs Interlayer Promotes Hole Extraction and Stability in Inverted CsPbI2.85Br0.15 Perovskite Solar Cells
Source: Molecules. 2025 Aug 29;30(17):3535. doi: 10.3390/molecules30173535 (PMC12430350; doi:10.3390/molecules30173535)
Supplement: Supplementary file 1 [file molecules-30-03535-s001.zip › molecules-3824628-supplementary.pdf]

Buried SWCNTs Interlayer Promotes Hole Extraction and Stability in Inverted $CsPbI_{2.85}Br_{0.15}$ Perovskite Solar Cells

Fangtao Yu [1,†], Dandan Chen [1,†], He Xi [1,*], Wenming Chai [2], Yuhao Yan [1], Weidong Zhu [2], Dazheng Chen [2], Long Zhou [1], Yimin Lei [1,*] and Chunfu Zhang [2,*]

[1] School of Advanced Materials and Nanotechnology, State Key Laboratory of Wide Bandgap Semiconductor Devices and Integrated Technology, Xidian University, Xi'an 710071, China
[2] Faculty of Integrated Circuit, Xidian University, Xi'an 710071, China

*Correspondence: hxi@xidian.edu.cn (He Xi); cfzhang@xidian.edu.cn (Chufu Zhang); leiym@xidian.edu.cn (Yimin Lei)

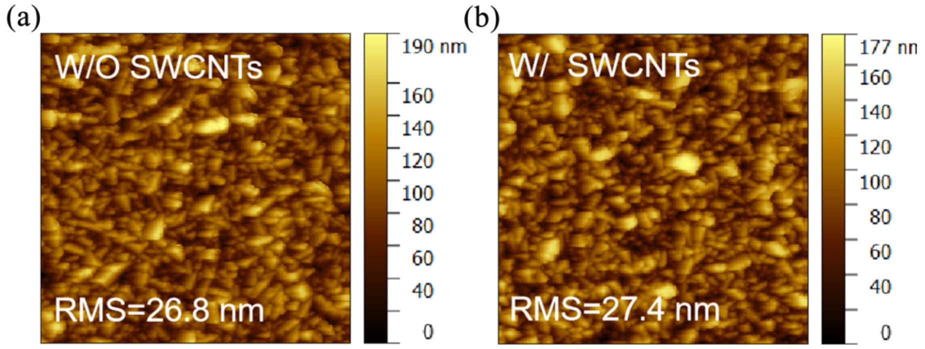

Figure S1. AFM images of MeO-2PACz films (a) without and (b) with SWCNTs modification.

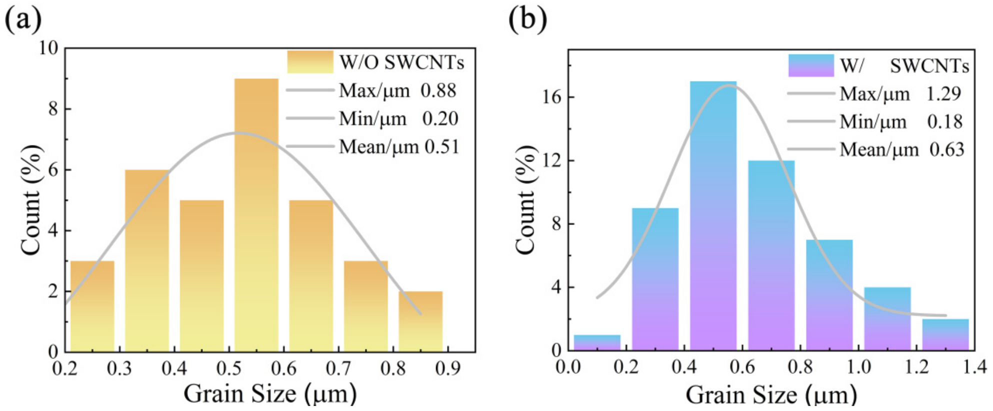

Figure S2. Statistical distribution of grain sizes in perovskite films (a) without and (b) with SWCNTs modification.

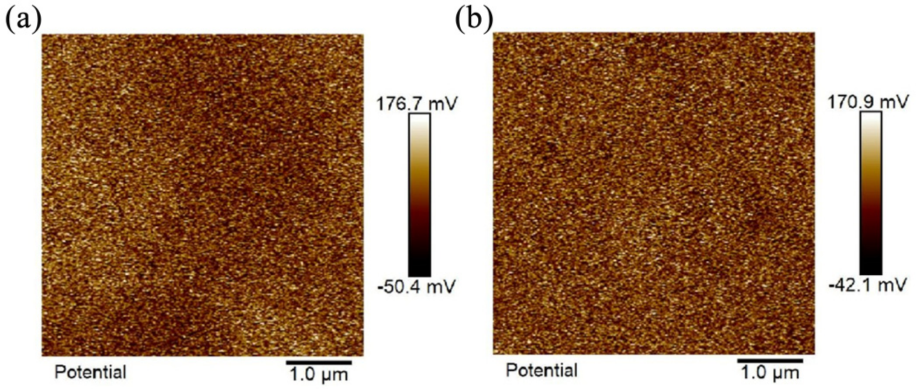

Figure S3. KPFM images of (a) pristine and (b) SWCNTs-modified perovskite films.

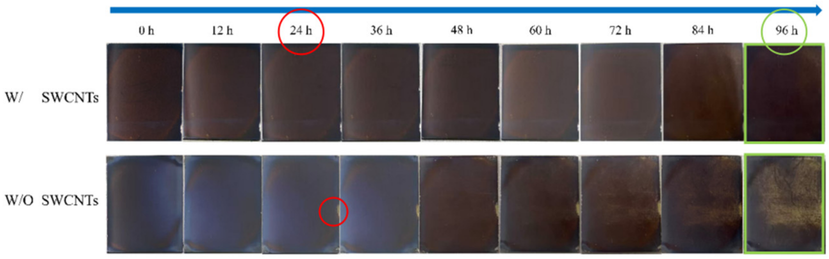

Figure S4. Degradation comparison of $CsPbI_{2.85}Br_{0.15}$ films with and without SWCNTs modification after 96 h exposure to ambient air (30 ~ 40% RH).

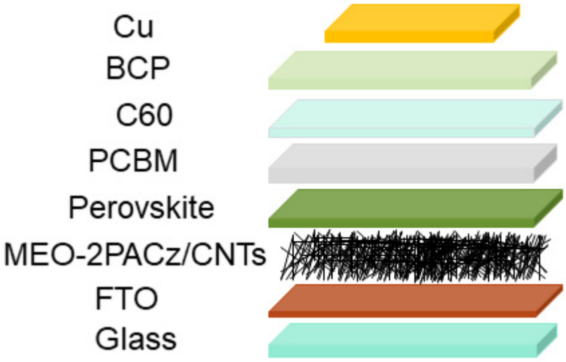

Figure S5. Device architecture of the p-i-n structured device.

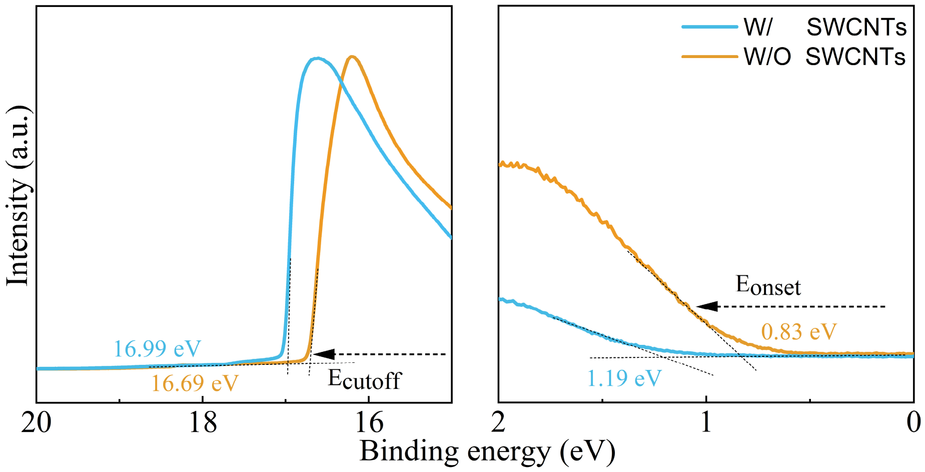

Figure S6. UPS spectra of MeO-2PACz films with and without SWCNTs modification, along with the estimated E_{onset} and E_{cutoff} values obtained from linear fittings.

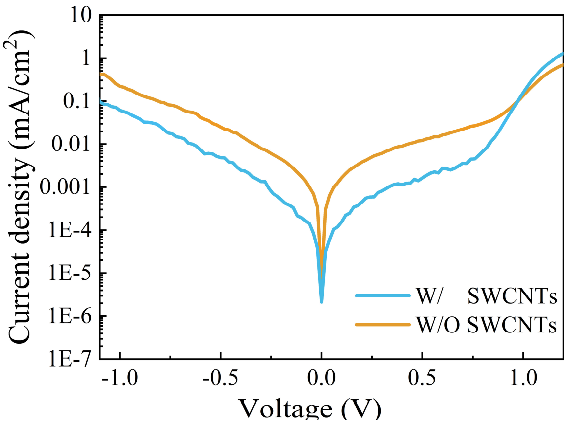

Figure S7. Dark current characteristics of devices with and without SWCNTs modification.

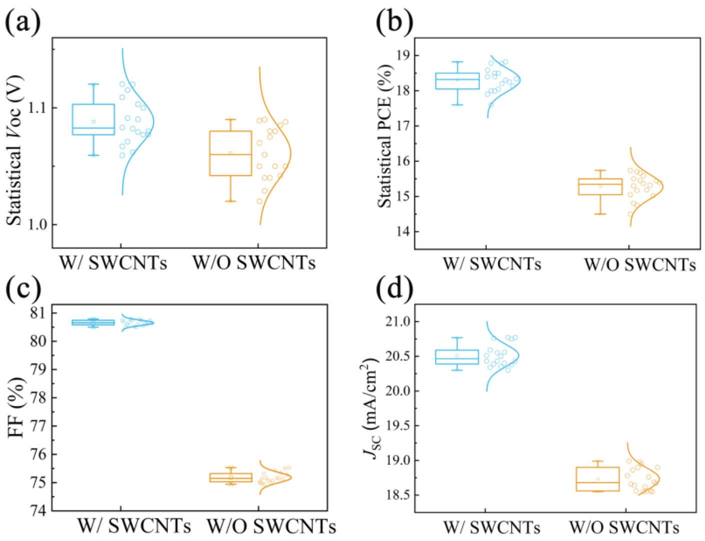

Figure S8. Statistical photovoltaic parameters of the control and SWCNTs-modified PSCs: (a) V_{oc}, (b) PCE, (c) FF and (d)J_{sc}.

Table S1. TRPL parameters of $CsPbI_{2.85}Br_{0.15}$ PSCs with and without SWCNTs modification.

Sample	τ_{ave} (ns)	τ_1 (ns)	A_1	τ_2 (ns)	A_2
W/O SWCNTs	8.94	4.32	0.97	22.93	0.06
W/ SWCNTs	6.49	3.30	0.75	15.48	0.06

Table S2. The parameters of the best-performing control devices ($CsPbI_{2.85}Br_{0.15}$) in the two studies.

Samples	V_{OC} (V)	J_{SC} (mA cm^{-2})	FF (%)	PCE (%)
W/O CsF	1.06	20.02	76.10	16.15
W/O SWCNTs	1.09	19.09	75.66	15.74

Table S3. Photovoltaic performance parameters of the best-performing $CsPbI_{2.85}Br_{0.15}$ PSCs without and with SWCNTs modification.

samples	V_{OC} (V)	J_{SC} (mA cm^{-2})	FF (%)	PCE (%)
W/O SWCNTs	1.09	19.09	75.66	15.74
W/ SWCNTs	1.12	20.83	80.81	18.78
